# CT trachea surface roughness is associated with chronic obstructive pulmonary disease symptoms

**DOI:** 10.1093/radadv/umae002

**Published:** 2024-03-19

**Authors:** Jason T Bartlett, James C Hogg, Jean Bourbeau, Wan C Tan, Miranda Kirby

**Affiliations:** Department of Physics, Toronto Metropolitan University, Toronto, ON, M5B 2K3, Canada; Center for Heart, Lung Innovation, University of British Columbia, Vancouver, BC, V6Z 1Y6, Canada; McGill University Health Centre, Montreal Chest Institute of the Royal Victoria Hospital, Montreal, QC, H4A 3J1, Canada; Respiratory Epidemiology and Clinical Research Unit, Research Institute of McGill University Health Centre, Montreal, QC, H4A 3S5, Canada; Center for Heart, Lung Innovation, University of British Columbia, Vancouver, BC, V6Z 1Y6, Canada; Department of Physics, Toronto Metropolitan University, Toronto, ON, M5B 2K3, Canada

**Keywords:** COPD, CT imaging, trachea abnormalities, fractal analysis, symptoms

## Abstract

**Background:**

Trachea structural abnormalities occur in patients with chronic obstructive pulmonary disease (COPD), yet there are few methods for quantifying trachea surface topology.

**Purpose:**

To develop a method to quantify trachea surface roughness on CT imaging and investigate the association with airflow limitation and symptoms in COPD.

**Materials and Methods:**

Participants from the multicenter prospective Canadian Cohort Obstructive Lung Disease study between 2009 and 2015 underwent CT imaging and analysis. Established CT measurements included: tracheal index (TI), defined as the smallest ratio of coronal-to-sagittal trachea diameter, low attenuation areas below –950 HU, and wall thickness of a theoretical 10-mm airway. Trachea surface roughness shape (SR_S_) was calculated as the percent fraction of the measurement box filled by the surface mesh. Multivariable regression models were used to determine association for CT measurements with forced expiratory volume in 1 second (FEV_1_) and forced vital capacity (FVC), and Medical Research Council dyspnea scale (MRC)≥3, adjusting for covariates.

**Results:**

A total of 1253 participants (mean age, 66 ± 10 years; 727 men) from 9 centers were investigated: *n* = 267 never smokers, *n* = 369 ever smokers, *n* = 352 mild COPD, and *n* = 265 moderate-to-severe COPD. There were no differences between groups for age or race (*P* < .05). In models including SR_S_ and TI, a 1-standard deviation (SD) increase in SR_S_ was independently associated with a 0.11-SD decrease in FEV_1_ (β = –0.11; *P* < .001) and a 0.16-SD decrease in FEV_1_/FVC (β = –0.16; *P* < .001); a 1-point increase in SR_S_ was associated with a 13% increased likelihood of MRC ≥ 3 (odds ratio = 1.13; *P* = .003). In models including SR_S_, low attenuation areas below –950 HU and wall thickness of a theoretical 10-mm airway, a 1-SD increase in SR_S_ was associated with a 0.21-SD decrease in FEV_1_ (β = –0.21; *P* < .001) and a 0.13-SD decrease in FEV_1_/FVC (β = –0.13; *P* < .001); a 1-point increase in SR_S_ was associated with a 12% increased likelihood of MRC ≥ 3 (odds ratio = 1.12; *P* = .006).

**Conclusion:**

Increased trachea surface shape roughness is independently associated with worse airflow and increased symptom burden in COPD.

**Abbreviations**

BMI = body mass index, CAT = COPD assessment, COPD = chronic obstructive pulmonary disease, FEV_1_ = forced expiratory volume in 1 second, FVC = forced vital capacity, MRC = Medical Research Council, Pi10 = wall thickness of a theoretical 10-mm airway, SR = surface roughness, TI = tracheal index, TLC = total lung capacity

**Summary**

A novel trachea lumen surface roughness measurement developed using fractal analysis was independently associated with worse airflow limitation and increased symptom burden in chronic obstructive pulmonary disease.

**Key Results**

- In models including the trachea surface roughness and trachea narrowing measurements, increased roughness was independently associated with reduced pulmonary function (*P* < .001), and only surface roughness was associated with increased presence of dyspnea (*P* = .003).
- In models including CT emphysema and airway inflammation measures, increased trachea surface roughness was independently associated with reduced pulmonary function (*P* < .001) and increased presence of dyspnea (*P* = .006).

## Introduction

Dyspnea, wheeze and cough are hallmark symptoms in patients with chronic obstructive pulmonary disease (COPD),^1^ and presence of symptoms is related to more rapid disease progression and increased mortality.^2^^,^^3^ Potential causes of dyspnea may include inspiratory muscle weakness and/or different forms of airway impedance, such as chronic bronchitis or tracheomalacia.^4^ Wheezing is caused by narrowing or obstruction of the airways, occurring throughout the airway tree.^5^ Chronic cough in COPD may be caused by mechanical factors, including attempts to clear lower airway mucus,^6^ and heighten cough sensitivity because of acute inflammation of epithelial layers within the central airways.^7^ Furthermore, lower airway remodeling and emphysema on CT imaging has been associated with both increased dyspnea^8^ and chronic cough,^8^^,^^9^ whereas only airway remodeling is associated with wheeze.^8^ However, the relationship between structural changes in the trachea and COPD symptoms is not well understood.

Airflow simulation studies of the central airways show the trachea surface structure has an impact on air flowing into and out of the lungs, with increased surface roughness resulting in increased airflow turbulence and particle deposition.^10^ Histopathological studies have demonstrated that chronic inflammation and remodeling observed in the peripheral airways also occurs in the trachea and main bronchi.^11^^,^^12^ In terms of CT studies, tracheal abnormalities, as measured by the trachea index (TI), have been associated with airflow limitation.^13^ However, the CT TI measurement uses only a single CT section representing the narrowest region of the trachea to quantify morphology and provides no information about the overall shape of the trachea surface. Furthermore, investigations focused on trachea topology and its impact on symptom burden in COPD patients has been limited.^14^

The objective of this study was to develop a novel methodology to quantify the entirety of the trachea lumen by measuring surface topology and roughness using fractal analysis^15^ in a population-based study of mainly mild COPD participants.^16^ Fractal analysis can be used to analyze the roughness of natural surfaces. As a surface becomes more contorted and rougher in shape, its dimensional value increases.^17^ We hypothesize that the trachea lumen surface roughness in individuals with COPD will be higher than those without COPD and be significantly associated with airflow limitation. Further, we hypothesize that trachea surface roughness in COPD will be significantly associated with worse symptoms, including dyspnea, wheeze, and cough.

## Materials and methods

### Study participants

**Figure 1.**
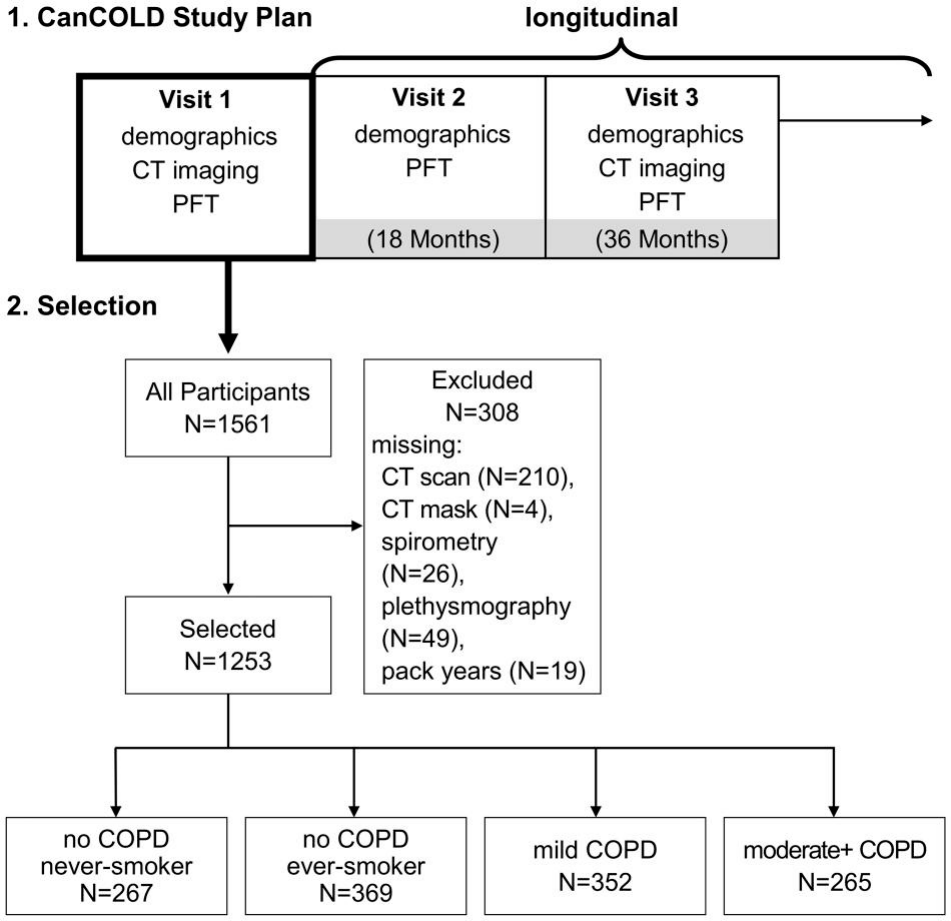
Flowchart of Canadian Cohort of Obstructive Lung Disease (CanCOLD) study plan and participants selected for trachea surface roughness analysis. PFT = pulmonary function test.

Participants aged >40 years were selected from the Canadian Cohort of Obstructive Lung Disease (CanCOLD)^16^ multicenter population-based study from 2009 to 2015. The study was approved by each site’s institution review board; all study participants provided written and informed consent. COPD status was defined as forced expiration volume in 1 second/forced vital capacity (FEV_1_/FVC) <0.70. Participants were categorized as never smokers and ever smokers (current or ex-smokers) without COPD, and Global Initiative for Chronic Obstructive Lung Disease I (mild) and Global Initiative for Chronic Obstructive Lung Disease II+ (moderate+) COPD.^18^ CanCOLD is a longitudinal study with follow-up visits conducted every 18 months for selected data collection (Figure 1). From the initial visit 1, 1561 participants were included in our cross-sectional analysis; 308 were excluded based on missing; CT imaging/masks, spirometry/plethysmography measurements; or smoking history; leaving 1253 participants. Further details of the study have been previously described.^16^

### Pulmonary function tests

Pulmonary function was assessed using spirometry and reporting the FEV_1_, FVC, and forced expiratory flow between 25% and 75% of the FVC (FEF_25–75%_).^18^^,^^19^ The total lung capacity (TLC) measurements were also obtained using whole-body plethysmography.^20^

The health status of participants was assessed using the COPD assessment (CAT) score, and a cutoff of CAT ≥ 10 was defined as having a significant health impact.^18^^,^^21^ Dyspnea was assessed using the Medical Research Council (MRC) dyspnea scale that grades self-reported breathlessness, where 1 is being out of breath only when strenuously exercising and 5 is equivalent to being too breathless to leave the house. A cutoff of MRC ≥ 3 was defined as having clinically significant shortness of breath^18^^,^^22^; a secondary lower cutoff of MRC ≥ 2 was also used to account for the overall mildness of the CanCOLD cohort. Finally, whether a participant had a recurrent wheeze or a persistent cough was recorded. Health impact, dyspnea, MRC ≥ 2, wheeze, and cough were recorded as binomial data.

### Image acquisition and analysis

CT imaging was acquired at full inspiration from apex to base of the lung. The image acquisition parameters used were: 120 kVp, 40 mAs, 0.5-second gantry rotation, pitch of 1.25, and 1 mm slice thickness, as previously described.^16^

CT analysis was performed using VIDA Diagnostics Apollo 2.0 software (VIDA Diagnostics, Inc, Coralville, IA, USA) for lung volume and airway segmentation. CT measurements included the wall thickness of a theoretical 10-mm airway (Pi10)^9^ and low attenuation areas of the lung below –950 Hounsfield units (LAA_950_).^23^ The TI measurement^24^ was the smallest cross-sectional coronal/sagittal diameter located on the trachea between the topmost region of the lung and the region located 2 cm above the carina. CT total lung volume (TLV_CT_) was also measured.

### Trachea lumen surface roughness

**Figure 2.**
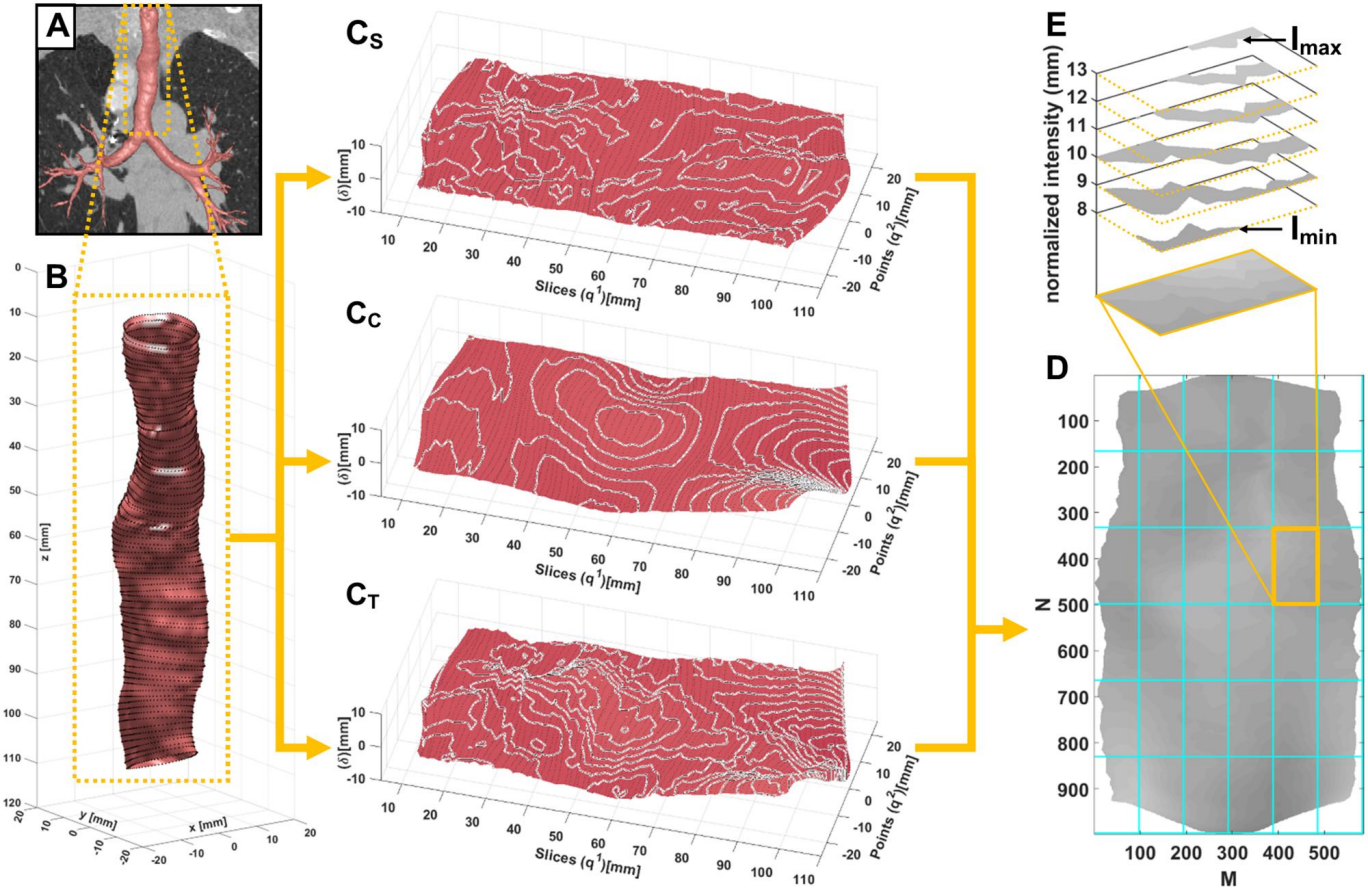
Trachea lumen surface extraction and roughness quantification workflow. (A) Segmented CT airway tree; (B) interpolated three-dimensional trachea surface reference points; (C) topological shape [S], curvature [C], and total [T] surface plots; (D) two-dimensional grayscale height map; (E) box counting with isolated gray levels.

All methods were implemented using MATLAB (MATLAB 2021, Natick, MA, USA: The MathWorks Inc.). The code and relevant scripts for surface extraction and fractal measurement are available on GITHUB at https://github.com/JasonBartlett/CTSurfaceRoughness. The detailed methods can be found in the online supplement. Figure 2 shows an overview of the surface extraction and roughness quantification using fractal dimension analysis. In brief, from the CT airway tree segmentation (Figure 2A), the CT trachea lumen surface was isolated starting at ∼5 mm below the top slice and ending at the carina (Figure 2B). The surface of the trachea lumen was mapped using three-dimensional reference points from which topological surfaces plots were generated^25^ consisting of the surfaces shape (Figure 2C), its curvature (Figure 2C), and a combined shape and curvature total surface (Figure 2C). The surfaces plots were converted into grayscale height maps (Figure 2D) in which the difference between each gray level was proportional to a 1-mm difference in height. Using the Integer Ratio Differential Box Counting Method^26^ (Figure 2E), each surface’s fractal dimension was then calculated and converted into surface roughness (SR) percentages. Higher SR values indicate increased surface roughness.

### Statistical analysis

All statistical analysis was performed using IBM SPSS Statistics Version 28 (Armonk, NY, USA). Univariate comparisons between never smokers, ever smokers, mild COPD, and moderate+ COPD participant group’s for demographics, pulmonary functions, symptoms, and CT measurements were performed using analysis of variance with Bonferroni post hoc comparisons. For categorical measurements, chi-square with Fisher exact tests were performed. For adjusted CT trachea measurements (SR, TI), analysis of covariance was performed. Correlations between trachea lumen SR and CT parameters and participant demographics were performed using a univariate Pearson’s correlation or independent *t*-tests. Multivariable linear regression models were used to determine associations between pulmonary function test and CT measurements. To determine independent associations between symptoms and CT measurements multivariable logistical regressions models were performed. All analysis of covariance and regression models were adjusted for age, sex, body mass index (BMI), pack years, smoking status, TLC, TLV_CT_/TLC, and CanCOLD site. Significance was defined as having a *P* value <.05. Bootstrapping (*n* = 1000) was applied to all SR_S_ regression models and Holm-Bonferroni corrections were applied to all regression models.

## Results

### Participants characteristics

CanCOLD participants recruited from 9 sites (Figure 1), their demographics, pulmonary function test, and symptom measurements among no COPD never smokers (*n* = 267), no COPD ever smokers (*n* = 369), mild COPD (*n* = 352), and moderate+ COPD (*n* = 265) groups are shown in Table 1. Of the selected participants who were identified as ever smokers, there were *n* = 682 ex-smokers and *n* = 184 current smokers. No COPD ever smoker participants had slightly higher BMI (*P* < .05) than those with mild COPD. There were fewer females in the mild COPD group compared with no COPD never/ever smoker (*P* < .05) and moderate+ COPD (*P* < .05) groups. Participants with moderate+ COPD had increased smoking pack-years compared with no COPD ever smokers (*P* < .05) and those with mild COPD (*P* < .05). There were fewer ex-smokers in both mild and moderate COPD (*P* < .05) groups than in the no COPD ever smoker group, and the moderate+ COPD group had more current smokers than the no COPD ever smoker (*P* < .05) and mild COPD (*P* < .05) groups. The breakdown for participant group populations for each of the CanCOLD study centers can be found in Table S1.

**Table 1.** Participant demographics, pulmonary function tests, and symptom scores.

Parameter (±SD unless specified)	All participants	No COPD		COPD	
		Never smoker	Ever smoker	Mild	Moderate+
	(*n* = 1253)	(*n* = 267)	(*n* = 369)	(*n* = 352)	(*n* = 265)
Demographics					
Age, y	66 (10)	66 (10)	66 (9)	68 (10)	67 (11)
BMI, kg/m^2^	27.5 (5.0)	27.2 (5.0)	28.1 (5.1)	27.1 (4.4)^b^	27.9 (5.5)
Sex female, *n* (%)	526 (42%)	139 (52)	153 (41)^a^	120 (32)^a^^,^^b^	115 (43)^c^
Race: White, *n* (%)	1191 (95%)	248 (93)	348 (94)	339 (96)	256 (97)
race: Other, *n* (%)	62 (5%)	19 (7)	21 (6)	13 (4)	9 (3)
Pack-years	17 (23)	–	19 (20)	18 (23)	28 (27)^b^^,^^c^
Ever smoker: ex-, *n* (%)	632 (50%)	–	300 (81%)	194 (55%)^b^	138 (52%)^b^
Current, *n* (%)	184 (15%)	–	69 (19%)	47 (13%)	68 (26%)^b^^,^^c^
Pulmonary function tests					
FEV_1_, L	2.57 (0.80)	2.77 (0.82)	2.79 (0.75)	2.74 (0.64)	1.84 (0.63)^a,b,c^
FEV_1_, % pred	92 (20)	103 (17)	99 (17)	96 (11)^a,b^	65 (12)^a,b,c^
FVC, L]	3.72 (1.07)	3.58 (1.07)	3.65 (0.99)	4.26 (0.99)^a,b^	3.23 (0.98)^a,b,c^
FEV_1_/FVC, %	69 (10)	78 (5)	77 (5)	65 (5)^a,b^	57 (10)^a,b,c^
FEF_25-75%_, L	1.76 (1.04)	2.46 (0.93)	2.43 (0.97)	1.31 (0.48)^a,b^	0.74 (0.49)^a,b,c^
TLC, L	6.36 (1.42)	5.97 (1.39)	6.14 (1.27)	6.96 (1.42)^a,b^	6.27 (1.40)^a,c^
TLV_CT_/TLC	0.74 (0.12)	0.73 (0.14)	0.73 (0.11)	0.76 (0.11)^a,b^	0.75 (0.10)
Symptoms					
Health impact (CAT ≥ 10), *n* (%)	295 (27)	38 (14)	72 (20)	63 (18)	122 (46)^a,b,c^
Dyspnea (MRC ≥ 3), *n* (%)	69 (6)	7 (3)	14 (4)	8 (2)	40 (15)^a,b,c^
Wheeze, *n* (%)	337 (27)	34 (13)	78 (21)^a^	89 (25)^a^	136 (51)^a,b,c^
Cough, *n* (%)	195 (16)	19 (7)	48 (13)^a^	48 (14)^a^	79 (30)^a,b,c^

Abbreviations: BMI = body mass index; CAT = COPD Assessment Test; COPD = chronic obstructive pulmonary disease; FEF = forced expiratory flow; FEV_1_ = forced expiratory volume in 1 s; FEV_1_ [% pred] = Global Lung Initiative (GLI) % predicted FEV_1_; FVC = forced vital capacity; MRC = Medical Research Council Dyspnea Scale; other = African, Asian, Hispanic, unknown; TLC = total lung capacity; TLV_CT_ = total lung volume CT; SD = standard deviation. Significantly different (*P* < .05).

a Never smoker.

b Ever smoker.

c Mild COPD.

The participants with moderate+ COPD had worse FEV_1,_ FVC, FEV_1_/FVC, and FEF_25-75%_ than both no COPD ever/never smoker groups, and those with moderate+ COPD had worse lung function than the participants with mild COPD (*P* < .05). Participants with mild+ COPD had increased FVC and worse FEV_1_/FVC and FEF_25-75%_ than both no COPD ever/never smoker (*P* < .05) groups.

The moderate+ COPD participants had significantly higher prevalence of dyspnea and poorer health status than both no COPD ever/never smoker and mild COPD (*P* < .05) groups. The no COPD ever smokers and mild COPD group also reported increased presence of wheeze and cough compared with the no COPD never smoker group (*P* < .05).

### CT measurements and trachea roughness

Table 2 shows comparison of the participant groups for CT whole lung and trachea measurements. Both the mild and moderate+ participant groups had increased CT LAA_950_ when compared with no COPD never and ever smoker groups (*P* < .05). The no COPD ever smoker, mild COPD, and moderate+ COPD groups had increased Pi10 compared with the no COPD never smoker group (*P* < .05).

**Table 2.** Whole-lung and trachea measurements for COPD groups.

Parameter (±SD unless specified)	All participants	no COPD		COPD	
		Never smoker	Ever smoker	Mild	Moderate+
	(*n* = 1253)	(*n* = 267)	(*n* = 369)	(*n* = 352)	(*n* = 265)
Demographics					
LAA_950_ (%)	4.3 (4.6)	2.9 (3.0)	3.0 (3.1)	5.5 (4.9)^a^^,^^b^	6.0 (6.1)^a^^,^^b^
Pi10 (mm)	3.96 (0.16)	3.93 (0.15)	3.96 (0.15)^a^	3.96 (0.16)^a^	3.99 (0.17)^a^
Trachea Index					
TI (d_c_/d_s_)	0.77 (0.13)	0.80 (0.12)	0.79 (0.13)	0.76 (0.11)^a^^,^^b^	0.75 (0.14)^a^^,^^b^
Trachea SR parameters					
SR_S_ (%)	16 (4)	15 (4)	16 (4)	17 (4)^a^^,^^b^	17 (5)^a^^,^^b^
SR_C_ (%)	18 (6)	18 (6)	17 (6)	19 (6)	18 (6)

Abbreviations: d_c_/d_s_ = coronal/sagittal diameter; COPD = chronic obstructive pulmonary disease; LAA_950_ = low attenuation area of the lung values below –950 HU on full-inspiration CT; Pi10 = the square root of the airway wall area for a theoretical airway with 10-mm internal perimeter; SD = standard deviation; SR = surface roughness = % fraction of measurement box filled by surface volume; SR_C_ = SR curvature; SR_S_ = SR shape; TI = tracheal index = the minimum ratio of trachea coronal diameter (d_c_) over sagittal diameter (d_s_). Significantly different (*P* < .05).

a Never smoker.

b Ever smoker.

**Figure 3.**
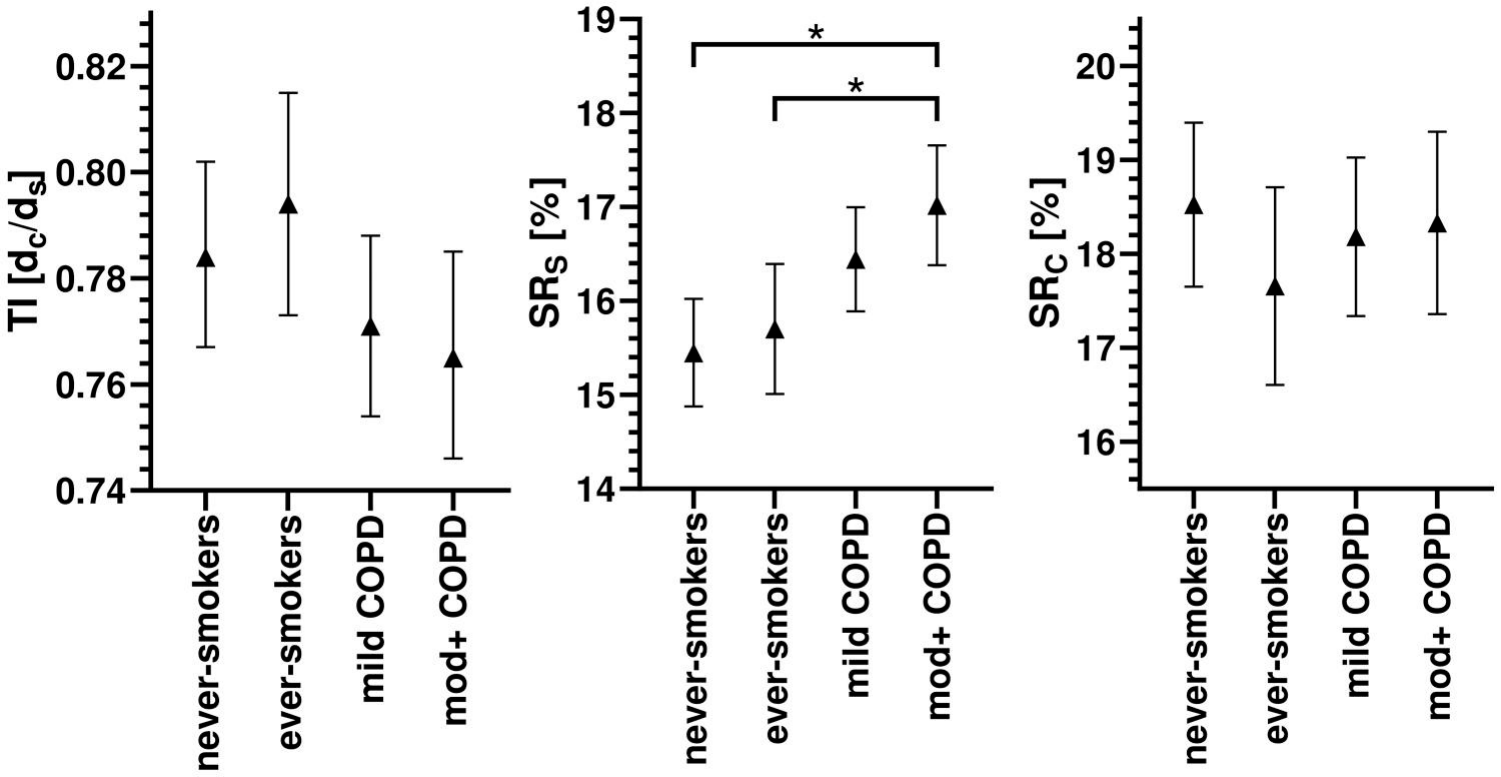
Comparison of tracheal index (TI) and surface roughness (SR) for GOLD groups after adjusting for covariates. Covariates used in the model were evaluated using population mean values: age = 66.63, BMI = 27.55, pack-years = 16.74, TLC = 6.361, TLV_CT_/TLC = .7395. Other factors include sex, smoking status, and study site. Stems indicate 95% confidence intervals: * = Significant difference (*P* < .05). BMI = body mass index; TI = minimum ratio of trachea coronal diameter over sagittal diameter; TLC = total lung capacity. GOLD (Global Initiative for Chronic Obstructive Lung Disease) criteria classify the severity of airflow limitation.

In the unadjusted analysis, TI was reduced and SR_S_ was increased in both COPD groups compared with the no COPD (*P* < .05) groups. After adjusting for covariates (Figure 3), TI was not significantly different between any groups. SR_S_ was significantly increased in the moderate+ COPD compared with both the no COPD ever/never smoker groups (*P* < .05). SR curvature (SR_C_) was not significantly different between any groups in unadjusted or adjusted analyses. An unadjusted analysis of SR total (SR_T_) can be found in Table S1.

**Figure 4.**
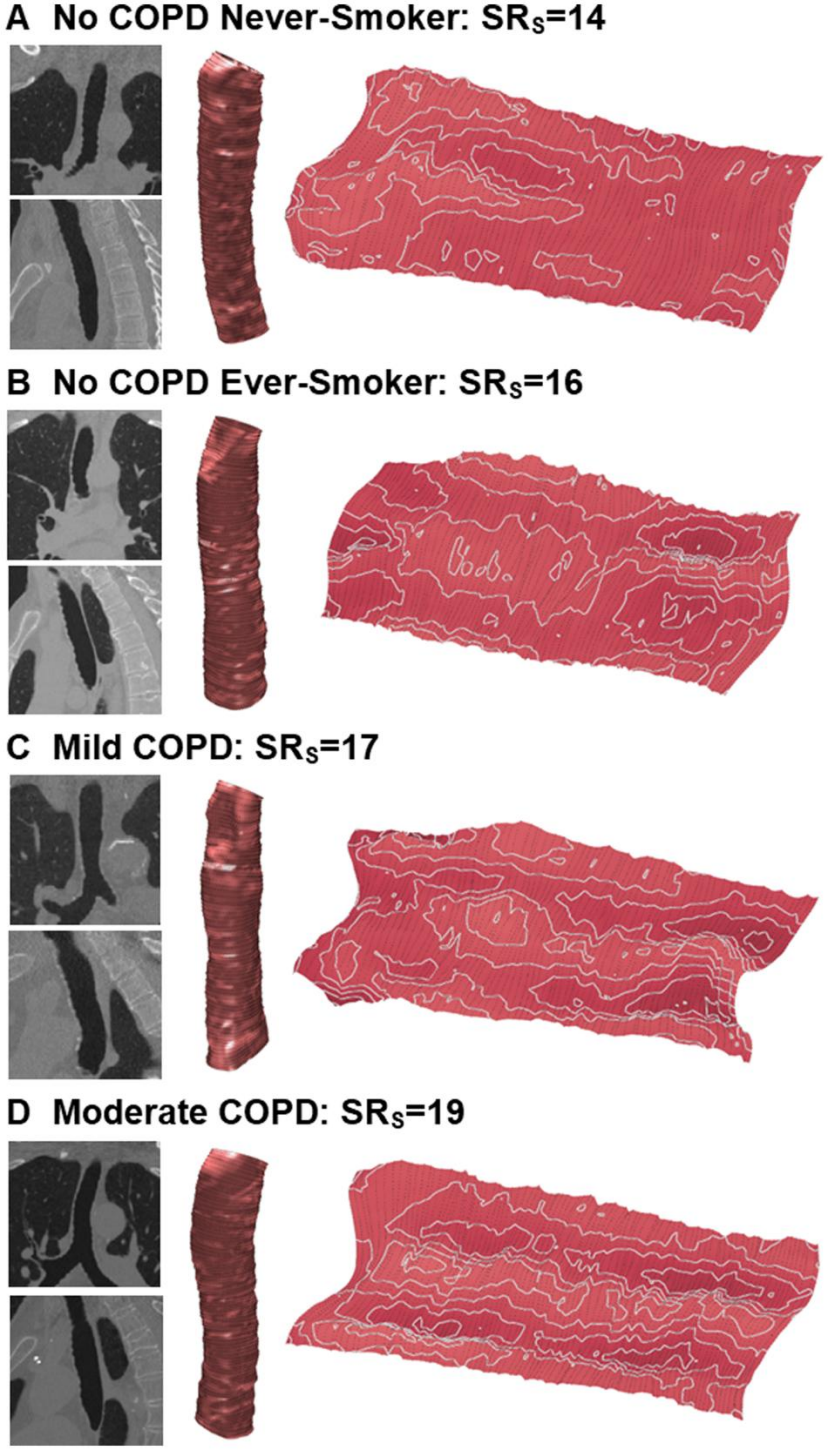
Trachea lumen surfaces (rolled and unrolled) for all participant groups. (A) Never smoker (62-year-old male, FEV_1_ = 3.58 L, FEV_1_/FVC = 76%, FEV_1_%pred = 98), (B) ever smoker (58-year-old male, FEV_1_ = 3.45 L, FEV_1_/FVC = 82%, FEV_1_%pred = 100). (C) Mild COPD (65-year-old male, FEV_1_ = 2.19 L, FEV_1_/FVC = 70%, FEV_1_%pred = 81), (D) moderate COPD (71-year-old male, FEV_1_ = 2.24 L, FEV_1_/FVC = 64%, FEV_1_%pred = 69). Forced expiratory volume in 1 second (FEV_1_) and forced vital capacity (FVC) and FEV_1_ percent of predicted value (FEV_1_%pred) are measured on pulmonary function tests. COPD = chronic obstructive pulmonary disease.

Figure 4 shows trachea lumen surfaces for representative participants in all groups. Qualitatively the trachea surface in the never smoker participants was rounder, with fewer and smaller deviations away from the central plane representing the ideal smooth surface. In the ever smoker participants, there is an increase presence of positive shape distortions or topological hills (lighter regions) and negative shape distortions or valleys (darker regions). In the COPD participants, steep slopes appear as the distance between the differences in height become smaller along with an increase in the number of overall surface deviations. Furthermore, in the moderate COPD participant, the formation of long continuous valley and hill regions are visible, indicating that the shape abnormally runs along the entire surface length.

### Multivariable linear regression models for CT SRs with pulmonary function measurements

The correlations between SR and CT trachea and whole lung measurements for all participants is shown in Table S2. SR_T_ was strongly correlated with SR_C_ (*r* = 0.84, *P* < .001) and SR_S_ (*r* = 0.75, *P* < .001); all other measurements were not strongly correlated (*r* < 0.60).

Table 3 shows multivariable linear regression models for FEV_1_, FEV_1_/FVC, and FEF_25-75_ with various combinations of CT whole lung and trachea measurements. For FEV_1_, when both SR_S_ and TI were included in the same model, only SR_S_ (β = –0.11, *P* < .001) was independently associated with FEV_1_. When SR_S_ and SR_C_ were included in the same model, again only SR_S_ (β = –0.14, *P* < .001) was associated with FEV_1_. Additionally, in a model for FEV_1_ with SR_S_, Pi10, and LAA_950_, the greatest standardized β coefficient was for LAA_950_ (β = –0.21, *P* < .001) followed by SR_S_ (β = –0.11, *P* < .001), whereas Pi10 was not significant.

**Table 3.** Multivariable linear regression models for SR_S_ (tracheal surface roughness shape) and CT measurements with pulmonary function.

Model	Std β	BCa 95% CI	*P* value
FEV_1_			
1: SR_S_	**–0.11** ^a^	**–**0.03 to **–**0.01	<.001
TI	0.06	0.06**–**0.68	.02
2: SR_S_	**–0.14** ^a^	**–**0.03 to **–**0.02	<.001
SR_C_	0.02	**–**0.003 to 0.007	.38
3: SR_S_	**–**0.11^a^	**–**0.03 to **–**0.01	<.001
LAA_950_	**–0.21** ^a^	**–**0.04 to **–**0.03	<.001
Pi10	**–**0.04	**–**0.45 to 0.02	.06
FEV_1_/FVC			
1: SR_S_	**–0.16** ^a^	**–**0.54 to **–**0.22	<.001
TI	0.07	0.28**–**10.38	.05
2: SR_S_	**–0.20** ^a^	**–**0.63 to **–**0.29	<.001
SR_C_	0.04	**–**0.03 to 0.15	.20
3: SR_S_	**–**0.13^a^	**–**0.45 to **–**0.17	<.001
LAA_950_	**–0.36** ^a^	**–**0.94 to **–**0.66	<.001
Pi10	**–**0.17^a^	**–**15.37 to **–**6.79	<.001
FEF_25-75_			
1: SR_S_	**–0.10** ^a^	**–**0.04 to **–**0.009	.002
TI	0.04	**–**0.13 to 0.85	.15
2: SR_S_	**–0.12** ^a^	**–**0.04 to **–**0.01	<.001
SR_C_	0.002	**–**0.009 to 0.01	.93
3: SR_S_	**–**0.09^a^	**–**0.04 to **–**0.007	.003
LAA_950_	**–0.22** ^a^	**–**0.06 to **–**0.04	<.001
Pi10	**–**0.07	**–**0.82 to **–**0.06	.02

All multivariable linear regression models included: age, sex, BMI, tobacco status, pack years. Bold = values with the largest Std β coefficient. Bootstrap n = 1000.

Abbreviations: BCa = bias corrected and accelerated; BMI, body mass index; FEF = forced expiratory flow; FEV_1_ = forced expiratory volume in 1 s; FVC = forced vital capacity; LAA_950_ = low attenuation area of the lung values below **–**950 HU on full-inspiration CT; Pi10 = the square root of the airway wall area for a theoretical airway with 10-mm internal perimeter; SR = surface roughness = % fraction of measurement box filled by surface volume; SR_C_ = SR curvature; SR_S_ = SR shape; TI (tracheal index)=the minimum ratio of trachea coronal diameter (d_c_) over sagittal diameter (d_s_); TLC = total lung capacity; TLV_CT_/TLC = CT total lung volume/TLC, and CanCOLD study center.

a Significant association (*P* < .05) after Holm-Bonferroni correction.

In the model for FEV_1_/FVC including TI, only SR_S_ (β = –0.16, *P* < .001) was independently associated with FEV_1_/FVC. In the model including SR_S_ and SR_C_, again only SR_S_ (β = –0.20, *P* < .001) was significantly associated with FEV_1_/FVC. When SR, Pi10, and LAA_950_ were included in a model for FEV_1_/FVC, the greatest standardized β coefficient was for LAA_950_ (β = –0.36, *P* < .001) followed by Pi10 (–0.17, *P* < .001), and then SR_S_ (β = –0.13, *P* < .001).

In the model for FEF_25-75_ including SR_S_ and TI, only SR_S_ (β = –0.10, *P* = .002) was independently associated, whereas TI was not. In the model including SR_S_ and SR_C_, again only SR_S_ (β = –0.12, *P* < .001) was significantly associated with FEF_25-75_. In the final model for FEF_25-75_ with SR_S_, Pi10, and LAA_950_, the greatest standardized β coefficient was for LAA_950_ (β = –0.22, *P* < .001) followed by SR_S_ (β = –0.09, *P* = .003), whereas Pi10 was not significant.

SR_C_ and SR_T_ were not significantly associated with FEV_1_, FEV_1_/FVC, or FEF_25-75_ in models with TI or Pi10 and LAA_950_ (Table S3).

### Multivariable logistic regression models for SR_S_ with COPD symptom scores

**Figure 5.**
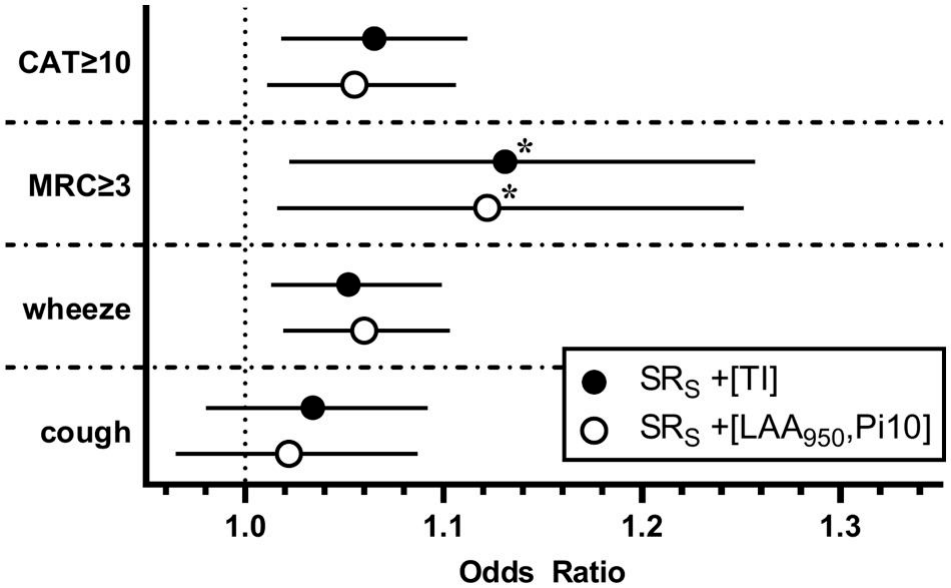
Odds ratios for SR_S_ with medium-high health impact CAT scores (CAT ≥ 10), presence of dyspnea (MRC ≥ 3), wheeze, and cough. COPD assessment (CAT) score and a cutoff of CAT ≥ 10 was defined as having a significant health impact. Base model adjusted by age, sex, BMI, tobacco status, pack years, TLC (total lung capacity), TLV_CT_/TLC (CT total lung volume/TLC), and CanCOLD site. CAT = COPD Assessment Test; LAA950 = low attenuation area of the lung values below –950 HU on full-inspiration CT; MRC = Medical Research Council Dyspnea Scale; Pi10 = the square root of the airway wall area for a theoretical airway with 10-mm internal perimeter; SR = surface roughness equals % fraction of measurement box filled by surface volume; SR_S_ = SR shape; TI = tracheal index is the minimum ratio of trachea coronal diameter (dc) over sagittal diameter (ds); *Significant association based on Holm-Bonferroni correction. Odds ratios and significance for TI, Pi10, and LAA_950_ can be found in Table S4.

Figure 5 shows the separate multivariable logistic regression models for COPD symptoms with SR_S_; the full multivariable regression models for TI, SR_C_, SR_T_, LAA_950_, and Pi10 can be found in Table S4. In a model including SR_S_ and TI, a 1-point increase in SR_S_ was significantly associated with a 13% increased likelihood of MRC ≥ 3 (OR = 1.13, *P* = .003). In a model that included SR_S_ and whole-lung CT LAA_950_ and Pi10, a 1-point increase of SR_S_ was significantly associated with a 12% increased likelihood of having a score of MRC ≥ 3 (OR = 1.12, *P* = .006). After using a Holm-Bonferroni correction, no CT trachea parameters were significantly associated with CAT ≥ 3, wheeze, and cough in any of the models.

Multivariable models including SR_C_ and SR_T_ are shown in Table S5. SR_C_ and SR_T_ were not associated with any symptom scores. A sensitivity analysis for multivariable regression models using MRC ≥ 2 can be found in Table S3; after the Holm-Bonferroni correction, there was no significant association with SR_S_.

## Discussion

In a population-based study of mainly mild COPD participants, trachea lumen surface shape roughness (SR_S_) was increased in participants with COPD compared with those without COPD and was associated with worse lung function and increased COPD symptom burden, independent of SR_C_ and whole-lung CT emphysema and airway disease measurements. Furthermore, our findings indicate that SR_S_ measurements provide trachea topological information related to lung function and COPD symptoms that are not captured by the TI.

Previous studies assessed trachea abnormalities using the CT TI measurement. A small study (*n* = 59) by Muro *et al.*^27^ showed no difference in CT TI measurements between COPD and non-COPD patients, and no correlation with pulmonary function. However, a larger study (*n* = 386) by Eom *et al.* did report differences for TI measurements between those with and without COPD, and correlations with spirometry. The discrepancy may stem from the smaller sample size in the study by Muro *et al.,*^27^ and the inability of the TI measurement to fully capture trachea shape abnormalities. In contrast, our findings in a mild COPD cohort demonstrates that the novel CT SR_S_ measurement was better able to quantify differences in trachea surface abnormalities between non-COPD and COPD groups than TI measurements. The SR_S_ measurement considers how the entire trachea surface is distorted, resulting in a more complete characterization of changes to its overall surface topology, even in those with mild COPD.

The SR_S_ measurement was associated with COPD symptoms, as measured by dyspnea, independent of TI, and whole-lung LAA_950_ and Pi10 measurements. Gallardo *et al.*^24^ included TI along with LAA_950_ and Pi10 in a classification model for COPD severity groups and showed TI was not significant. In contrast, our findings show that in a multivariable regression model with SR_S_, LAA_950_, and Pi10, SR_S_ was independently associated with pulmonary function. Moreover, the SR_S_ measurement had the greatest relative contribution in the model for dyspnea. These results emphasize the importance of tracheal surface topological measurements, and not simply trachea diameter, in explaining COPD severity and symptoms.

Finally, we demonstrated that tracheal lumen shape abnormalities on CT differentiate participants with and without COPD, and are associated with worse lung function and symptoms, independent of trachea curvature. Few studies have investigated how trachea geometry such as shape and curvature influences airflow. A small airflow simulation study demonstrated abnormal trachea curvature independent of stenosis resulted in pressure loss and increased turbulence in the lower regions of the trachea.^28^ Another study showed rougher surfaces resulted in an increase in edge shear forces and increased turbulence compared with the ideal smooth surfaced model.^29^ Our findings align with the latter, emphasizing trachea surface shape as a dominant factor.

Trachea abnormalities in patients with COPD may arise from various factors, including chronic airway inflammation resulting in central airway narrowing,^11^ pressure differences between the central airways and the pleural cavity resulting from lung hyperinflation that elongates the trachea while reducing the trachea’s coronal-sagittal ratio,^30^ and excessive coughing causing damage to the cartilaginous rings.^31^ The presence of paratracheal paraseptal emphysema was also shown to be associated with abnormal trachea features.^32^ Although the underlying causes of the trachea abnormalities are beyond the scope of this study, the trachea measurements were only weakly correlated with emphysema and remained significant in models adjusted by emphysema, suggesting that trachea topology abnormalities are independently associated with lung function and symptoms in COPD.

There are limitations of this study that should be considered. Trachea analysis was performed on inspiratory images, and as such the impact of tracheal abnormalities that require both inspiratory-expiratory scans, such as expiratory central airway collapse,^33^ were not investigated. Comparing inspiratory and expiratory trachea surfaces with one another requires that the region of interest be spatially matched; therefore, a more extensive method development is needed that is beyond the scope of this study. Future research will focus on investigating trachea abnormalities quantified with both full-expiration and full-inspiration images. Additionally, although a standard image acquisition protocol was used across CanCOLD, studies have shown that different CT models and reconstruction kernels have a direct impact on Hounsfield unit values.^34^^,^^35^ Different segmentation approaches may also impact SR values. Future studies should investigate the impact of various segmentation approaches and different acquisition parameters (eg, reconstruction kernel, slice thickness) on trachea surface roughness measurements.

Nevertheless, our findings indicate that quantifying tracheal abnormalities adds to existing quantitative CT measures. Future studies should investigate the clinical relevance of trachea analysis and its potential role in clinical practice; for example, establishing normative SR_S_ values for male and female populations to aid in identification of abnormal trachea surface features. CanCOLD is a population-based cohort study and is therefore an ideal population for establishing normative reference ranges and prediction models, as previously described.^36^ Future studies should also investigate the association between SR_S_ and longitudinal decline in lung function, or evaluate the efficacy of interventions,^37^ to provide a greater understanding of the role this measurement may have in clinical management. Prediction models incorporating these CT measurements could also be used for early detection of individuals at risk of rapid disease progression to facilitate early treatment options. Prediction models incorporating trachea analysis, along with existing quantitative measures, could also be used clinically in settings where chest imaging is acquired for other purposes, such as lung cancer screening trials. SR_S_ values could be used to help identify people who have undiagnosed trachea surface abnormalities and could be at risk of having COPD. Leveraging quantitative CT measures extracted from the trachea may provide important prognostic information that can be used to improve detection and monitoring of lung disease.

In conclusion, a novel approach to quantify trachea lumen surface topology and roughness on CT images was developed using fractal dimensional analysis. SR_S_ measurements were increased in COPD, and associated with worse airflow limitation and COPD symptoms, independent of existing trachea measurements or whole lung emphysema and airway remodeling measures. These findings suggest the CT SR_S_ measurement is an important new biomarker that can help to better understand the impact that trachea abnormities have on airflow limitation and symptom burden in those with COPD.

## Supplementary Material

umae002_Supplementary_Data

## Data Availability

The data underlying this article were provided by CanCOLD by permission. Data will be shared on request to the corresponding author with permission of CanCOLD.
