# Supplementary material for: CT trachea surface roughness is associated with chronic obstructive pulmonary disease symptoms
Source: Radiol Adv. 2024 Mar 19;1(1):umae002. doi: 10.1093/radadv/umae002 (PMC12429235; doi:10.1093/radadv/umae002)
Supplement: umae002_Supplementary_Data [file umae002_Supplementary_Data.docx]

**CT Trachea Surface Roughness is Associated with Chronic Obstructive Pulmonary Disease Symptoms**

Original Research

**Summary Statement (27/30 words):** A novel trachea lumen surface roughness measurement developed using fractal analysis was independently associated with worse airflow limitation and increased symptom burden in chronic obstructive pulmonary disease.

**Key Results (62/75 Words)**

1. In models including the trachea surface roughness and trachea narrowing measurements, increased roughness was independently associated with reduced pulmonary function (p<0.001), and only surface roughness was associated with increased presence of dyspnea (p=0.003).
2. In models including CT emphysema and airway inflammation measures, increased trachea surface roughness was independently associated with reduced pulmonary function (p<0.001) and increased presence of dyspnea (p=0.006).

# SUPPLEMENTARY MATERIAL

# METHODS

## Trachea Surface Extraction Full Method

All methods were implemented using MATLAB (MATLAB (2021), Natick, Massachusetts: The MathWorks Inc.). The VIDA trachea surface 3D point cloud was extracted for each participant ($T=[X_{s,p},Y_{s,p},Z_{s,p}]$) where s=slice number and p=perimeter point along with its center-line ($C=[\vec{x}_{s,c},\vec{y}_{s,c,},\vec{z}_{s,c}]$) and rescaled using the CT volume voxel dimensions $(\vec{v}={[x}_{dim}{,y}_{dim},z_{dim}])$. Point clouds T and C were then rigidly rotated and translated using an affine matrix ($A$); such that their central axis as drawn between the central point aligned with the top of the lung and the top of the aortic arch were parallel to the z axis and aligned to $x=0,y=0$(eq 1,2).

$C'=\left[ C\times\vec{v} \right][A]$(1)

$T'=\left[ T\times\vec{v} \right][A]$(2)

A perfectly smooth trachea surface point cloud $P^{S}=[X_{s,p}^{P},Y_{s,p}^{P},Z_{s,p}]$) was then generated based on point cloud $T^{'}$.

$X_{s,p}^{P'}=\cos\left( \theta_{s,p} \right)*R$(3)

$Y_{s,p}^{P'}=\sin\left( \theta_{s,p} \right)*R$(4)

Where $\theta_{s,p}$ is the aligned surface points location in arc radians, $R=\bar{P}_{Mid}/2\pi$ is the radius of the ideal cylinder and $\bar{P}_{Mid}$ is the average of the perimeters of the middle 3^rd^ trachea slices. Finally, a third surface was generated by aligning the center of each slice of $P^{S}$ such that $C^{'}(\vec{x}_{s,c},\vec{y}_{s,c,})=[0,0]$ creating a perfectly smooth cylinder $P^{C}$.

Each trachea surface profile mesh plots were then unrolled and mapped into a 3D surface mesh $S_{s,p}=({q_{1}}_{s,p},{q_{2}}_{s,p}, \delta_{s,p})$ based on an approach by Qiao, L *et al.,*(25) using curvilinear coordinates as follows

${q_{1}}_{s,p}=z_{s,p}^{'}$;(5)

${q_{2}}_{s,p}=\sin^{-1}(\sqrt{d^{2}-{\Delta z}^{2}}/2R)2R$;(6)

$\delta_{s,p}=\cos\left( \varphi\right)h$(7)

Where ${q_{1}}_{s,p}$ (eq 5) is the surface points location along the z axis; ${q_{2}}_{s,p}$ (eq 6) is the surface points equivalent arc length location on the surface being extracted, $d=\left\| T_{s,p}^{'}-T_{s,p+1}^{'} \right\|_{2}$ is the Euclidian distance between two adjacent surface slice points, $\Delta z=z_{s,p}^{'}-z_{s,p+1}^{'}$ is the vertical distance between two adjacent surface slice points; $\delta_{s,p}$ is the total deviation of the surface point in the x-plane from the surface of the surface being compared to, where $\varphi$ is the angle between the center point of the smooth surface and the point on the surface profile being mapped, and $h$ is the distance between equivalent surface points. The $\delta_{s,p}$ for the surface mesh shape ($M_{S}$) profile was generated by comparing the surface differences between $T^{'}$and $P^{S}$ (figure S1A), the surface mesh curvature ($M_{C}$) profile was generated by comparing $P^{C}$to $P^{S}$ (figure S1B ), and the surface mesh total ($M_{T}$)was generated by comparing $T^{'}$to $P^{C}$ (figure S1C ). The resulting $M(\delta_{s,p})$ are then projected onto a 2D 64 grayscale level image of 𝑀×𝑁 size where

$M = \left\lceil\max\left( {q_{2}}_{s,p} \right)-\min\left( {q_{2}}_{s,p} \right) \right\rceil\times10$;(8)

$N = \left\lceil\max\left( {q_{1}}_{s,p} \right)-\min\left( {q_{1}}_{s,p} \right) \right\rceil\times10$;(9)

and the intensity or grayscale level ($I$) of each pixel is proportional to its normalized surface height rounded to the nearest mm distance.

$I\propto round\left( \left[ \delta_{s,p}-\min\left( \delta_{s,p} \right) \right] \right)+1$.(10)

The fractal dimension (D) for each trachea surface was then found using the Integer Ratio Differential Box Counting Method (IRDBC) (26). The 𝑀×𝑁 grayscale surface was divided up into a series of $m\times n$ sized boxes whose dimensions were integer ratios of $r:$=$\left[ 2,\ldots,Q \right]$where $Q=\left\lfloor\sqrt[3]{MN} \right\rfloor$. Values of $r$ were selected to maximize surface coverage and minimize distance to $log\left( r \right)$=[0.5,1,1.5,…,5]. The size of each box was then determined by eqs (11,12),

$m\left( r \right)=floor\left\lfloor M/r \right\rfloor$.(11)

$n\left( r \right)=floor\left\lfloor N/r \right\rfloor$.(12)

The box count $n_{r}\left( i,j \right)$ of each $m$×$n$ box was then determined using eq(13)

$n_{r}\left( i,j \right)=\left( \frac{I_{max}\left( i,j \right)-I_{min}\left( i,j \right)}{G/r}+1 \right)\times\left( \frac{A\left( i,j \right)-nan}{mn} \right)$ ;(13)

where $G=64$ is the maximum gray levels used in the image and $I_{max}\left( i,j \right)$ and $I_{min}\left( i,j \right)$ are the maximum and minimum grey level intensities of the $m$×$n$ box being investigated, $A\left( i,j \right)$ is the actual box area, $mn$ is its expected area, and $nan$ is the number of empty cells in the box area being investigated. Finally, the sum $N_{r}\left( r \right)$ eq (14) is the sum of all boxes for each value of $r$,

$N_{r}\left( r \right)=\sum n_{r}\left( i,j \right)$;(14)

where the D eq (15) of each trachea surface is the value of the slope of a loglog plot

$D=\frac{\log\left( N_{r}\left( r \right) \right)}{log\left( r \right)}$.(15)

Finally, the surface roughness (SR) of each surface was found by calculating the difference between surface D and the expected value of a smooth surface (D=2) (eq 16)

$SR=\left( D-2 \right)*100$.(16)

# FIGURE LEGENDS

**Figure S1**: Trachea Lumen surface mesh profile extraction for, A) shape, B) curvature, and C) total mesh profile extractions. Black points represent surface being extracted; cyan points represent smooth surface being compared to.

**Supplemental Tables**

| Table S1: Supplemental Study Center Demographics, Symptom Scores, and Trachea Measurements | | | | | |
| --- | --- | --- | --- | --- | --- |
| Parameter  (±SD unless specified) | all  participants  (n=1253) | no COPD | | COPD | |
|  |  | never-smoker  (n=267) | ever-smoker  (n=369) | mild  (n=352) | moderate+ (n=265) |
| Demographics | | | | | |
| Vancouver | 363 (29%) | 58 (22%) | 105 (28%) | 119 (34%)^*^ | 81 (31%)^*^ |
| Montreal | 304 (24%) | 62 (23%) | 101 (27%) | 70 (20%)^†^ | 71 (27%) |
| Toronto | 50 (4%) | 11 (4%) | 10 (3%) | 20 (6%) | 9 (3%) |
| Halifax | 58 (5%) | 10 (4%) | 16 (4%) | 20 (6%) | 12 (5%) |
| Calgary | 116 (9%) | 35 (13%) | 34 (9%) | 30 (9%) | 17 (6%)^*^ |
| Ottawa | 84 (7%) | 19 (7%) | 17 (5%) | 28 (8%) | 20 (8%) |
| Kingston | 117 (9%) | 30 (11%) | 44 (12%) | 27 (8%) | 16 (6%)^*†^ |
| Quebec | 73 (6%) | 20 (7%) | 24 (7%) | 8 (2%)^*†^ | 21 (8%)^‡^ |
| Saskatoon | 88 (7%) | 22 (8%) | 18 (5%) | 30 (9%) | 18 (7%) |
| Symptoms | | | | | |
| MRC≥2, n (%) | 437 (35%) | 57 (21%) | 110 (30%)^*^ | 121 (34%)^*^ | 149 (56%)^*†‡^ |
| Trachea Surface Roughness (SR) Parameters | | | | | |
| SR_T_, [%] | 23 (5) | 22 (5) | 22 (5) | 23 (5)^*†^ | 23 (5)^*†^ |
| SD=standard deviation; MRC= Medical Research Council Dyspnea Scale; SR (surface roughness)=% fraction of measurement box filled by surface volume; SR_T_=SR total. Significantly different (p<0.05) from * never-smoker, † ever-smoker, ‡ mild COPD. | | | | | |

Table S2: Surface Roughness CT vs Trachea and Whole Lung CT Values: Univariate Correlations

| Variable [95% C.I.] | SR_S_ | SR_C_ | SR_T_ |
| --- | --- | --- | --- |
| SR_C_ | 0.44* [0.40, 0.49] |  |  |
| SR_T_ | 0.75* [0.72, 0.77] | 0.84* [0.82, 0.85] |  |
| TI | -0.55* [-0.59, -0.51] | -0.27* [-0.32, -0.21] | -0.39* [-0.44, -0.34] |
| LAA_950_ | 0.27* [0.22, 0.32] | 0.16* [0.10, 0.21] | 0.21* [0.16, 0.26] |
| Pi10 | 0.31* [0.26, 0.36] | 0.17* [0.11, 0.22] | 0.23* [0.18, 0.28] |
| SR (surface roughness)=% fraction of measurement box filled by surface volume; SRc=SR curvature; SR_T_=SR total; TI (tracheal index)=the minimum ratio of trachea coronal diameter (d_c_) over sagittal diameter (d_s_); LAA_950_=low attenuation area of the lung values below -950 HU on full-inspiration CT; Pi10=the square root of the airway wall area for a theoretical airway with 10mm internal perimeter. *=Significantly correlated (p<0.05). | | | |

| Table S3: Multivariable Linear Regression Model for SR_C_, SR_T_ and CT Measurements with Pulmonary Function | | | |
| --- | --- | --- | --- |
| Model | Std β | 95% CI [LB,UB] | p-value |
| FEV_1_ |  |  |  |
| 1: SR_C_ | -0.01 | [-0.006, 0.004] | 0.79 |
| TI | **0.10*** | [0.36, 0.87] | <0.001 |
| 2: SR_C_ | -0.003 | [-0.005, 0.005] | 0.89 |
| LAA_950_ | **-0.22*** | [-0.04, -0.03] | <0.001 |
| Pi10 | -0.06* | [-0.51, -0.09] | 0.005 |
| 1: SR_T_ | -0.04 | [-0.01, 0.001] | 0.09 |
| TI | **0.09*** | [0.31, 0.82] | <0.001 |
| 2: SR_T_ | -0.04 | [-0.01, <0.001] | 0.07 |
| LAA_950_ | **-0.21*** | [-0.04, -0.03] | <0.001 |
| Pi10 | -0.05* | [-0.49, -0.07] | 0.009 |
| FEV_1_/FVC | | | |
| 1: SR_C_ | 0.002 | [-0.09, 0.10] | 0.94 |
| TI | **0.13*** | [5.55, 14.87] | <0.001 |
| 2: SR_C_ | 0.02 | [-0.06, 0.11] | 0.56 |
| LAA_950_ | **-0.37*** | [-0.95, -0.71] | <0.001 |
| Pi10 | -0.19* | [-16.18, -8.84] | <0.001 |
| 1: SR_T_ | -0.05 | [-0.21, 0.02] | 0.10 |
| TI | **0.11*** | [4.59, 14.07] | <0.001 |
| 2: SR_T_ | -0.04 | [-0.17, 0.04] | 0.21 |
| LAA_950_ | **-0.37*** | [-0.94, -0.70] | <0.001 |
| Pi10 | -0.19* | [-15.87, -8.51] | <0.001 |
| FEF_25-75_ | | | |
| 1: SR_C_ | -0.02 | [-0.01, 0.006] | 0.49 |
| TI | **0.08*** | [0.19, 1.10] | 0.006 |
| 2: SR_C_ | -0.01 | [-0.01, 0.007] | 0.62 |
| LAA_950_ | **-0.23*** | [-0.07, -0.04] | <0.001 |
| Pi10 | -0.08* | [-0.90, -0.15] | 0.007 |
| 1: SR_T_ | -0.04 | [-0.02, 0.004] | 0.20 |
| TI | 0.07 | [0.13, 1.06] | 0.01 |
| 2: SR_T_ | -0.03 | [-0.02, 0.004] | 0.25 |
| LAA_950_ | **-0.23*** | [-0.06, -0.04] | <0.001 |
| Pi10 | -0.08* | [-0.89, -0.13] | 0.009 |
| All multivariable linear regression models included: age, sex, BMI, tobacco status, pack years, TLC=total lung capacity, TLV_CT_/TLC=CT total lung volume/TLC, and CanCOLD study center. FEV_1_=forced expiratory volume in one second; FVC=forced vital capacity; FEF=Forced Expiratory Flow; SR (surface roughness)=% fraction of measurement box filled by surface volume; SRc=SR curvature; SR_T_=SR total; TI (tracheal index)=the minimum ratio of trachea coronal diameter (d_c_) over sagittal diameter (d_s_); LAA_950_=low attenuation area of the lung values below -950 HU on full-inspiration CT; Pi10=the square root of the airway wall area for a theoretical airway with 10mm internal perimeter. *=Significant association (p<0.05) after Holm-Bonferroni correction; Bold= values with the largest Std β coefficient. | | | |

| Table S4: Multivariable Binary Logistical Regression Model for SR_S_ and CT Measurements with COPD Symptoms | | | |
| --- | --- | --- | --- |
| Model | Odds Ratio | BCa 95% CI [LB,UB] | p-value |
| health impact [CAT≥10] | | | |
| 1: SR_S_ | 1.07 | [1.02, 1.11] | 0.01 |
| TI | 0.77 | [0.19, 3.07] | 0.73 |
| 2: SR_S_ | 1.06 | [1.01, 1.11] | 0.01 |
| LAA_950_ | **1.08*** | [1.04, 1.13] | <0.001 |
| Pi10 | 2.72 | [0.67, 13.14] | 0.14 |
| dyspnea [MRC ≥3] |  |  |  |
| 1: SR_S_ | **1.13*** | [1.02, 1.26] | 0.003 |
| TI | 0.61 | [0.06, 6.61] | 0.68 |
| 2: SR_S_ | **1.12*** | [1.02, 1.25] | 0.006 |
| LAA_950_ | 1.10* | [1.03, 1.23] | 0.005 |
| Pi10 | 11.27 | [0.44, 333.62] | 0.08 |
| Wheeze | | | |
| 1: SR_S_ | 1.05 | [1.01, 1.10] | 0.01 |
| TI | 0.37 | [0.10, 1.33] | 0.14 |
| 2: SR_S_ | 1.05 | [1.01, 1.09] | 0.01 |
| LAA_950_ | 1.05* | [1.01, 1.10] | 0.006 |
| Pi10 | 5.88 | [1.64, 27.88] | 0.01 |
| Cough |  |  |  |
| 1: SR_S_ | 1.03 | [0.98, 1.09] | 0.20 |
| TI | 0.38 | [0.06, 2.17] | 0.27 |
| 2: SR_S_ | 1.04 | [0.99, 1.10] | 0.11 |
| LAA_950_ | 1.02 | [0.98, 1.07] | 0.29 |
| Pi10 | 2.13 | [0.46, 8.56] | 0.31 |
| MRC ≥2 | | | |
| 1: SR_S_ | 1.05 | [1.01, 1.10] | 0.01 |
| TI | 1.10 | [0.25, 4.18] | 0.89 |
| 2: SR_S_ | 1.04 | [1.00, 1.07] | 0.08 |
| LAA_950_ | 1.07* | [1.03, 1.13] | <0.001 |
| Pi10 | **11.86*** | [2.56, 76.10] | <0.001 |
| All multivariable linear regression models included: age, sex, BMI, tobacco status, pack years, TLC, TLV_CT_/TLC, and study center. Bca=bias-corrected and accelerated bootstrap interval; SR (surface roughness)=% fraction of measurement box filled by surface volume; SRs=SR shape; TI (tracheal index)=the minimum ratio of trachea coronal diameter (d_c_) over sagittal diameter (d_s_); LAA_950_=low attenuation area of the lung values below -950 HU on full-inspiration CT; Pi10=the square root of the airway wall area for a theoretical airway with 10mm internal perimeter. *=Significant association (p<0.05) after Holm-Bonferroni correction; Bold=values with the largest Std β coefficient. Bootstrap n=1000 | | | |

| Table S5: Multivariable Binary Logistical Regression Model for SR_C_, SR_T_ and CT Measurements with COPD Symptoms | | | |
| --- | --- | --- | --- |
| Model | Odds Ratio | 95% CI [LB,UB] | p-value |
| health impact [CAT≥10] | | | |
| 1: SR_C_ | 1.00 | [0.98, 1.03] | 0.88 |
| TI | 0.36 | [0.10, 1.29] | 0.12 |
| 2: SR_C_ | 1.00 | [0.98, 1.03] | 0.81 |
| LAA_950_ | **1.08*** | [1.04, 1.12] | <0.001 |
| Pi10 | 3.50 | [0.93, 13.18] | 0.06 |
| 1: SR_T_ | 1.02 | [0.99, 1.05] | 0.31 |
| TI | 0.41 | [0.11, 1.50] | 0.18 |
| 2: SR_T_ | 1.02 | [0.99, 1.05] | 0.29 |
| LAA_950_ | **1.08*** | [1.04, 1.12] | <0.001 |
| Pi10 | 3.31 | [0.88, 12.47] | 0.08 |
| dyspnea [MRC ≥3] |  |  |  |
| 1: SR_C_ | 1.02 | [0.97, 1.07] | 0.41 |
| TI | 0.13 | [0.01, 1.36] | 0.09 |
| 2: SR_C_ | 1.02 | [0.98, 1.07] | 0.36 |
| LAA_950_ | **1.11*** | [1.05, 1.17] | <0.001 |
| Pi10 | 14.39 | [1.22, 169.74] | 0.03 |
| 1: SR_T_ | 1.04 | [0.98, 1.10] | 0.17 |
| TI | 0.17 | [0.02, 1.82] | 0.14 |
| 2: SR_T_ | 1.04 | [0.99, 1.11] | 0.13 |
| LAA_950_ | **1.11*** | [1.05, 1.16] | <0.001 |
| Pi10 | 13.15 | [1.11, 155.68] | 0.04 |
| Wheeze | | | |
| 1: SR_C_ | 0.99 | [0.97, 1.02] | 0.56 |
| TI | **0.19*** | [0.06, 0.63] | 0.007 |
| 2: SR_C_ | 0.99 | [0.97, 1.02] | 0.58 |
| LAA_950_ | 1.06* | [1.02, 1.10] | 0.003 |
| Pi10 | **7.66*** | [2.17, 27.04] | 0.002 |
| 1: SR_T_ | 1.02 | [0.99, 1.05] | 0.24 |
| TI | 0.23 | [0.07, 0.78] | 0.02 |
| 2: SR_T_ | 1.02 | [0.99, 1.05] | 0.19 |
| LAA_950_ | 1.06* | [1.02, 1.10] | 0.003 |
| Pi10 | **6.86*** | [1.94, 24.18] | 0.003 |
| Cough |  |  |  |
| 1: SR_C_ | 1.03 | [1.00, 1.07] | 0.04 |
| TI | 0.28 | [0.06, 1.22] | 0.09 |
| 2: SR_C_ | 1.04 | [1.00, 1.07] | 0.03 |
| LAA_950_ | 1.03 | [0.99, 1.07] | 0.21 |
| Pi10 | 2.38 | [0.53, 10.75] | 0.26 |
| 1: SR_T_ | 1.03 | [1.00, 1.07] | 0.07 |
| TI | 0.33 | [0.07, 1.47] | 0.15 |
| 2: SR_T_ | 1.04 | [1.00, 1.08] | 0.04 |
| LAA_950_ | 1.03 | [0.99, 1.07] | 0.23 |
| Pi10 | 2.25 | [0.50, 10.18] | 0.30 |
| All multivariable linear regression models included: age, sex, BMI, tobacco status, pack years, TLC, TLV_CT_/TLC, and study center. SR (surface roughness)=% fraction of measurement box filled by surface volume; SRc=SR curvature; SR_T_=SR total; TI (tracheal index)=the minimum ratio of trachea coronal diameter (d_c_) over sagittal diameter (d_s_); LAA_950_=low attenuation area of the lung values below -950 HU on full-inspiration CT; Pi10=the square root of the airway wall area for a theoretical airway with 10mm internal perimeter. *=Significant association (p<0.05) after Holm-Bonferroni correction; Bold=values with the largest Std β coefficient. | | | |
